# New hair, new rash: is there a correlation?

**DOI:** 10.1097/JW9.0000000000000271

**Published:** 2026-06-25

**Authors:** Mallory A. Von Lotten, Tiffany T. Mayo

**Affiliations:** a Department of Medicine, University of Alabama at Birmingham Heersink School of Medicine, Birmingham, Alabama; b Department of Dermatology, University of Alabama at Birmingham, Birmingham, Alabama

**Keywords:** allergic contact dermatitis, atopic dermatitis, lichen simplex chronicus, paraphenylenediamine, patch testing, scalp dermatitis, synthetic hair extensions

**What is known about this subject in regard to women and their families?**

- Synthetic hair extensions and some hair dyes can cause allergic contact dermatitis where hair rests on the skin (neck/hairline/ears); patch testing often implicates paraphenylenediamine and disperse dyes/acrylates.

**What is new from this article as messages for women and their families?**

- Lateral-neck dermatitis temporally linked to synthetic extensions with clinicopathologic correlation, improving after allergen avoidance.
- Practical counseling—including options that preserve protective styling (eg, untreated human hair, low-residue heat-resistant fibers)—with specific relevance for women with skin of color who frequently use these styles.

## Case summary

**Fig. 1.**
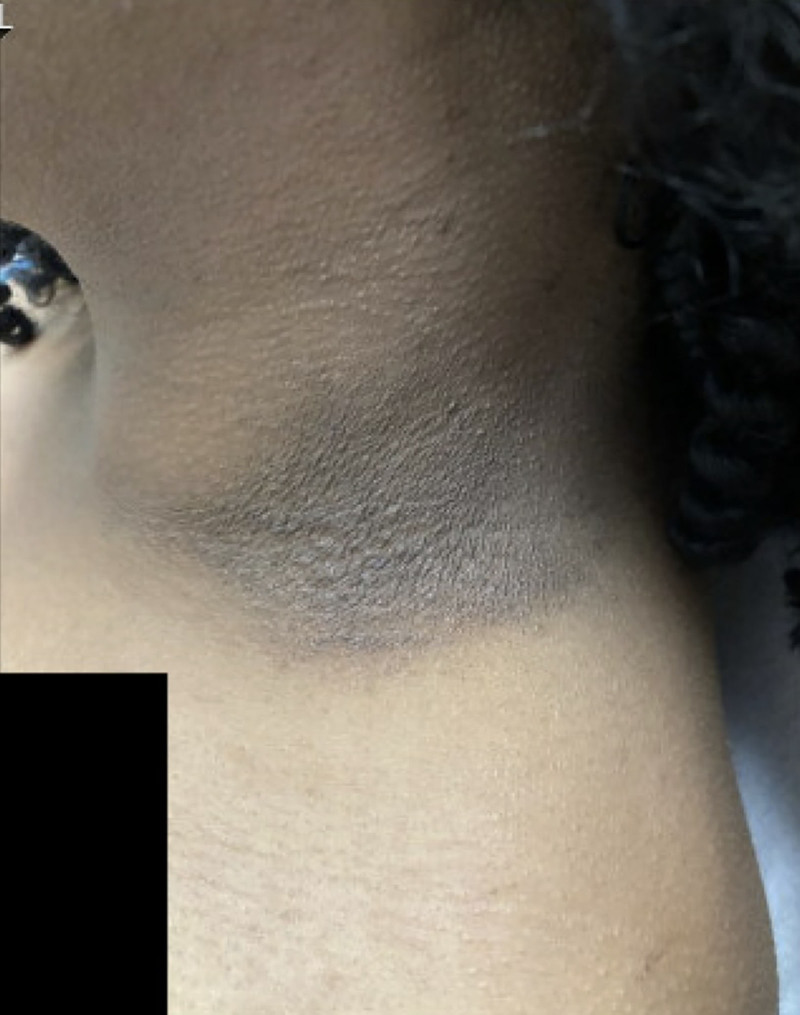
Lichenified plaques symmetrically distributed around the lateral necks, corresponding to areas in contact with synthetic hair extensions, highlighting the potential role of allergens and irritants.

A 37-year-old African American woman with Fitzpatrick skin type V and a history of atopic dermatitis presented with a persistent, pruritic rash on the neck accompanied by scalp irritation and hair thinning. Further history revealed that her symptoms worsened after each new hairstyle involving synthetic hair extensions. Examination showed hyperpigmented, lichenified plaques symmetrically distributed around the neck, periorbital regions, ears, and axillae (Fig. 1). The patient denied known contact triggers. She reported transient relief with triamcinolone 0.1% ointment but overall progression of pruritus and hyperpigmentation. Tacrolimus 0.1% ointment was prescribed twice daily, and patch testing was recommended to identify specific allergens. The patient was counseled to discontinue synthetic hair products and consider alternatives less likely to contain commonly encountered allergens, such as untreated human hair or heat-resistant, low-residue synthetic fibers to prevent recurrence.

This case underscores the importance of recognizing allergic contact dermatitis (ACD) related to synthetic hair ingredients and providing culturally competent counseling to reduce recurrence.

**Question #1: What is your diagnosis?**

A. Psoriasis
B. ACD
C. Tinea corporis
D. Seborrheic dermatitis
E. Lichen planus

**Correct Answer: B. ACD**

ACD is a delayed hypersensitivity reaction triggered by allergens such as paraphenylenediamine (PPD), commonly found in synthetic hair fibers and hair dyes. In contrast, psoriasis presents as well-demarcated plaques with silvery scale and may be associated with nail pitting or psoriatic arthritis. Tinea corporis typically appears as annular plaques with central clearing, while seborrheic dermatitis manifests as ill-defined erythematous patches with greasy scale. Lichen planus presents as polygonal, violaceous papules and plaques.

## Discussion

Contact dermatitis from synthetic hair is an increasing clinical concern, particularly among individuals who frequently use extensions, wigs, or weaves. Synthetic hair fibers often contain sensitizing compounds such as PPD, acrylates, methacrylates, and formaldehyde-releasing resins, all of which can elicit delayed-type hypersensitivity reactions. Acrylates and methacrylates—present in some fiber coatings and bonding adhesives—have been increasingly recognized as relevant allergens.^1,2^

While some allergens may impair the skin barrier and penetrate deeper into epidermal layers, barrier disruption does not occur with all exposures. This mechanism is particularly significant in individuals with atopic dermatitis, in whom preexisting barrier compromise enhances allergen penetration and immune activation.^3,4^

**Fig. 2.**
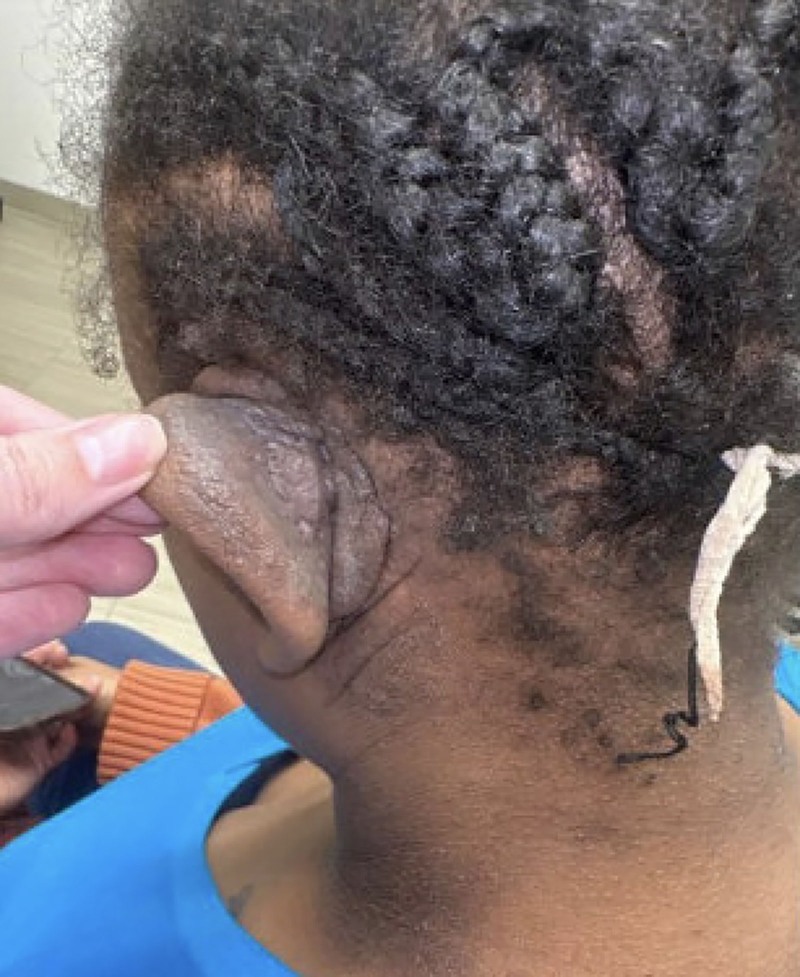
Severe lichenified plaques with hyperpigmentation and thickening involving the posterior auricular region, extending behind the ear in an area of prolonged synthetic hair contact. This highlights the potential for widespread allergic contact dermatitis in occluded regions.

Clinically, lesions typically occur along sites of direct contact—most often the lateral neck, ears, and periorbital regions—supporting a diagnosis of contact dermatitis (Fig. 2). The patient’s chronic lichenification reflected a combination of allergic inflammation and mechanical irritation from repetitive hair friction.

Despite the widespread use of synthetic hair, regulatory oversight of its chemical composition remains limited. Unlike cosmetics, hair extensions and weaves are not subject to strict FDA regulation, leading to inconsistent labeling and potential exposure to unlisted allergens.^5^ Increased transparency in product formulations and dermatologic advocacy for safer materials is essential to reducing ACD incidence.

Hair extensions and weaves are deeply integrated into hairstyling practices, particularly among African American women, making this issue both medical and cultural. Many individuals rely on synthetic hair for protective styling and self-expression. Dermatologists should provide culturally competent counseling and reinforce the importance of patch testing for those in whom ACD is suspected.

Diagnosis of synthetic hair–induced ACD requires a thorough clinical history and confirmatory patch testing.^6^ In addition to PPD, testing panels should include various types of acrylates, including methacrylates, and formaldehyde, to identify relevant allergens. Histopathology, when performed, may reveal spongiotic dermatitis with a lymphocytic infiltrate with eosinophils, consistent with ACD.^7^

Management begins with avoidance of the offending material and use of topical therapies to reduce inflammation and pruritus. Short courses of topical corticosteroids remain the first-line treatment for acute flares, providing faster symptomatic relief, particularly on the neck and ears. Calcineurin inhibitors, such as tacrolimus 0.1% ointment, serve as steroid-sparing agents suitable for sensitive areas after the acute phase improves or for more persistent dermatitis. Systemic corticosteroids are reserved for severe or refractory cases.

Preventive counseling is key: patients should be educated on allergen avoidance, safe styling techniques, and the importance of early evaluation for recurrent dermatitis. As synthetic hair use continues to rise, dermatologists play an essential role in raising awareness, advocating for safer product development, and addressing disparities in hair care–related dermatoses.^8,9^

**Question #2: Which of the following would be considered one of the first-line treatments for this patient?**

A. Oral antihistamines
B. Topical calcineurin inhibitors
C. Systemic corticosteroids
D. Ultraviolet B radiation phototherapy
E. Methotrexate

**Correct Answer: B. Topical calcineurin inhibitors**

Topical calcineurin inhibitors such as tacrolimus are effective steroid-sparing agents, making them ideal for delicate areas such as the neck and face and for long-term use. Patch testing remains essential for identifying allergens and guiding long-term management, while allergen avoidance is critical for preventing recurrence.

**Question #3: A patient develops pruritic, hyperpigmented plaques after using synthetic hair extensions. Patch testing reveals a strong positive reaction to a chemical found in hair dyes and textiles. Which allergen is the most likely culprit?**

A. Nickel sulfate
B. PPD
C. Fragrance mix
D. Neomycin sulfate
E. Lanolin

**Correct Answer: B. PPD.** PPD is a well-documented sensitizer found in synthetic hair, hair dyes, and textiles. It is a potent allergen known to induce a delayed-type hypersensitivity reaction resulting in pruritic, lichenified plaques.

## Conflicts of interest

T.T.M. serves as an investigator for Acelyrin, BMS, Galderma, Janssen, Lilly, Pfizer, Sanofi, and Boehringer Ingelheim. She has served as a consultant for Abbvie, Arcutis, BMS, Bodewell, Janssen, Lilly, Leo, Merck, Novartis, Pfizer, Takeda, and UCB. The other author has no conflicts of interest to disclose.

## Funding

None.

## Study approval

N/A.

## Author contributions

MAVL contributed to study conception and design, data collection, analysis and interpretation, and manuscript drafting. TTM contributed to study conception and design, study supervision, data interpretation, and critical revision of the manuscript. Both authors reviewed and approved the final manuscript.

## Patient consent

The authors obtained written consent from patients for their photographs and medical information to be published in print and online, and with the understanding that this information may be publicly available. Patient consent forms were not provided to the journal but are retained by the authors.
